# Fluid responsiveness prediction using Vigileo FloTrac measured cardiac output changes during passive leg raise test

**DOI:** 10.1186/s40560-016-0188-6

**Published:** 2016-10-06

**Authors:** Anton Krige, Martin Bland, Thomas Fanshawe

**Affiliations:** 1 Department of Anaesthesia and Critical Care, Royal Blackburn Hospital, Haslingden Road, Blackburn, UK; 2 Nuffield Department of Primary Care Health Sciences, University of Oxford, Oxford, UK

**Keywords:** Passive leg raising, Edwards Vigileo FloTrac monitoring, Fluid responsiveness, Cardiac output monitoring, Vasoplegic shock, Septic shock

## Abstract

**Background:**

Passive leg raising (PLR) is a so called self-volume challenge used to test for fluid responsiveness. Changes in cardiac output (CO) or stroke volume (SV) measured during PLR are used to predict the need for subsequent fluid loading. This requires a device that can measure CO changes rapidly. The Vigileo™ monitor, using third-generation software, allows continuous CO monitoring. The aim of this study was to compare changes in CO (measured with the Vigileo device) during a PLR manoeuvre to calculate the accuracy for predicting fluid responsiveness.

**Methods:**

This is a prospective study in a 20-bedded mixed general critical care unit in a large non-university regional referral hospital. Fluid responders were defined as having an increase in CO of greater than 15 % following a fluid challenge. Patients meeting the criteria for circulatory shock with a Vigileo™ monitor (Vigileo™; FloTrac; Edwards™; Lifesciences, Irvine, CA, USA) already in situ, and assessed as requiring volume expansion by the clinical team based on clinical criteria, were included. All patients underwent a PLR manoeuvre followed by a fluid challenge.

**Results:**

Data was collected and analysed on stroke volume variation (SVV) at baseline and CO and SVV changes during the PLR manoeuvre and following a subsequent fluid challenge in 33 patients. The majority had septic shock. Patient characteristics, baseline haemodynamic variables and baseline vasoactive infusion requirements were similar between fluid responders (10 patients) and non-responders (23 patients). Peak increase in CO occurred within 120 s during the PLR in all cases. Using an optimal cut point of 9 % increase in CO during the PLR produced an area under the receiver operating characteristic curve of 0.85 (95 % CI 0.63 to 1.00) with a sensitivity of 80 % (95 % CI 44 to 96 %) and a specificity of 91 % (95 % CI 70 to 98 %).

**Conclusions:**

CO changes measured by the Vigileo™ monitor using third-generation software during a PLR test predict fluid responsiveness in mixed medical and surgical patients with *vasopressor-dependent circulatory* shock.

**Electronic supplementary material:**

The online version of this article (doi:10.1186/s40560-016-0188-6) contains supplementary material, which is available to authorized users.

## Background

Circulatory insufficiency is common in critically ill patients and may lead to organ dysfunction. Increasing cardiac preload may increase stroke volume (SV) and consequently cardiac output (CO) and thus tissue perfusion [1]. However, it has been shown that only half of critically ill patients thought to be preload responsive, based on static predictors of preload, actually show an increase in SV, and therefore CO, following volume expansion (VE) [2, 3]. The administration of fluid in the group that is not preload responsive not only delays the appropriate management of their circulatory insufficiency but is also an independent predictor of delayed respiratory weaning and survival [4–11].

Functional haemodynamic monitoring has been shown to accurately predict fluid responsiveness but unfortunately several caveats, which include constant tidal volumes of adequate size and sinus rhythm, must be present [12–17]. Unfortunately, this precludes a large proportion of critically ill patients.

Passive leg raising (PLR), whereby the patients legs are transiently raised by 45°, and the torso flattened, results in a reversible flow of 150–300 ml of blood from the venous capacitance vessels of the lower body, to the thoracic compartment, a so called self-volume challenge [18–20]. Boulain et al. [21] first demonstrated the potential of this phenomenon to predict fluid responsiveness by measuring blood pressure changes during a PLR.

Subsequent studies have shown even greater accuracy, without the limitations of functional haemodynamic monitoring, using a variety of minimally invasive devices capable of rapid measurement of changes in flow or stroke volume during PLR. Examples include esophageal Doppler [22, 23], transthoracic Doppler ultrasound [24], transthoracic echocardiography (TTE) [25–29], pulse contour techniques [28, 30–33], bioreactance [34–36], end tidal carbon dioxide change [37] and bio-impedance cardiography [38]. These studies have been summarised in three recent systematic reviews [39–41], and all reported a high sensitivity regarding prediction of fluid responsiveness (all three reported the same pooled area under the receiver operating characteristic curve (AUC) of 0.95, with similar confidence intervals).

Only one of these [28] studies used the Vigileo™ monitor but with second-generation software.

The aim of this study was to assess the accuracy of the Vigileo™ monitor (Vigileo™; FloTrac; Edwards™; Lifesciences, Irvine, CA, USA) using third-generation software (version 3.02), in predicting preload responsiveness by measuring changes in CO during a PLR manoeuvre, in a mixture of medical and surgical critically ill patients with circulatory shock, with or without spontaneous breathing efforts or arrhythmias. In addition, we included an assessment of the accuracy of stroke volume variation (SVV) measured by the same device to predict responsiveness. We evaluated a cut point of 9.6 % at baseline (derived by Li et al. [42]), and the change in SVV during PLR were both evaluated as predictors of fluid responsiveness.

## Methods

### Patients

This prospective study was conducted on a mixed medical and surgical general critical care unit in a large non-university regional referral hospital. The study received research ethics committee approval and patients were enrolled following written informed consent.

### Inclusion criteria

Critical care patients, over 18 years of age, requiring a fluid challenge as decided by the attending critical care physician, were included. The research team had no influence over this decision, and patients were only approached for enrolment following the decision to administer a fluid challenge.

This decision was based on the presence of at least one clinical sign of inadequate tissue perfusion, i.e. (a) systolic blood pressure <90 mmHg (or a decrease of >50 mmHg in previously hypertensive patients) or the need for vasoconstrictor drugs (vasopressin or norepinephrine); (b) urine output <0.5 ml/kg/h for ≥2 h; (c) tachycardia (heart rate >100/min); or (d) presence of skin mottling.

The Vigileo system (Edwards Lifescience, Irvine, CA) with arterial pressure waveform analysis device via special blood flow sensor (FloTrac Sensor, Edwards Lifesciences, Irvine, CA) using third-generation software must already be in situ.

### Exclusion criteria

Any patients that were unable to perform PLR and who had any contraindications to fluid challenge, defined as life-threatening hypoxaemia, and evidence of blood volume overload and/or hydrostatic pulmonary oedema, were excluded.

### Measurements

All haemodynamic data was continuously recorded on a Draeger monitoring system and a Vigileo system (Edwards Lifesciences, Irvine, CA) using third-generation software (version 3.02).

### VigileoTM monitor measurements

The FloTrac transducer (FloTracTM, Edwards Lifesciences, Irvine, CA, USA) connected the indwelling arterial line to the VigileoTM System (Edwards Lifesciences, Irvine, CA, USA). This non-calibrated continuous CO monitor software analyses the arterial waveform with a frequency of a 100 Hz over 20 s. SV is calculated as *k* × pulsatility, where pulsatility is the standard deviation of arterial pressure over the preceding 20 s, and *k* is a factor which describes vascular compliance and resistance over the preceding 1 to 5 min, depending on the setting. This factor (so called proprietary Dynamic Tone Technology) is derived from a multivariate regression model taking into account Langewouter’s aortic compliance [43], mean arterial pressure (MAP), along with variance, skewness, and kurtosis of the arterial pressure wave [43, 44]. A greater number of hyperdynamic and vasodilated patients were incorporated into the algorithm database, and additional physiologically based variables were added to the algorithm’s vascular tone *k* factor in order to adjust automatically for hyperdynamic and vasodilated patients in the third-generation software update. This has increased the accuracy of SV measurement in vasodilated patients [45–47].

### Other measurements

The presence of any arrhythmias was recorded. Respiratory data was collated regarding the mode of ventilation, presence of spontaneous respiratory efforts, peak inspiratory pressures, plateau pressures, PEEP value and tidal volume. Dosages of any vasoactive drugs were recorded.

#### Study design

**Fig. 1:**
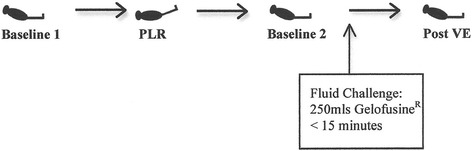
Study design. Patient positioning during the four stages of measurement as described

The study design is illustrated in Fig. 1. The following peak haemodynamic parameters were all recorded at each of the four stages in the protocol: heart rate (HR), mean arterial pressure (MAP), central venous pressure (CVP), cardiac index (CI), CO, SV, stroke volume index (SVI) and stroke volume variation (SVV). At the first baseline stage, measurements were taken while the patient was 45° semi-recumbent. During the next stage (PLR), the remote control of the bed was used to tilt the trunk to a horizontal position, and the legs were tilted 45° upwards. The patient was then returned to the 45° semi-recumbent position until the haemodynamic parameters stabilised to similar values as the original baseline. These values were recorded as representing the second baseline stage. Following this, a VE of 250 ml of Gelofusine^R^ was administered at the maximum rate allowed by the volumetric pump (within 15 min). The final stage, denoted as post volume expansion (PVE), occurred immediately following the fluid challenge, when all haemodynamic parameters were recorded once again.

#### Statistical analysis

Continuous data are reported as mean and standard deviation or median and inter-quartile range. A change in CO from baseline of >15 and <15 % following the volume challenge was used to classify the patients as ‘responders’ and ‘non-responders’, respectively [3]. Measured variables were then compared between responders and non-responders using *t* tests, chi-squared tests or Fisher’s exact test for low counts, as appropriate for the data type, and reported as *p* values. The positively skewed variables of time to admission, tidal volume and norepinephrine dose were analysed after log-transforming the original data values. A receiver operating characteristic (ROC) curve was used to measure the sensitivity and specificity of the PLR test to predict responders to the fluid challenge.

*p* values of less than 0.05 were considered to be statistically significant. Statistical analysis was performed using R version 2.15.2 [48].

## Results

### Patient characteristics

Thirty-seven patients with CO monitoring in situ and meeting clinical criteria for a fluid challenge were initially included and four were subsequently excluded. Three had different diagnoses (cardiogenic shock with blood volume overload which was an exclusion criteria), and one had missing baseline and PLR CO data. The data from the remaining 33 patients who received a fluid challenge were analysed. Patient characteristics are summarised in Table 1 and were similar between groups. The majority (80 %), overall and in each group, were on a controlled mode of ventilation, with similar tidal volumes between groups. Cardiac arrhythmias were present in 9 % of all patients and balanced between the groups. The aetiology of circulatory insufficiency was septic shock in the majority (82 %) and severe systemic inflammatory response syndrome (SIRS) in the remainder.

**Table 1:** Patient characteristics

	All	Responders	Non-responders	
	*n* = 33	*n* = 10	*n* = 23	*p*
Age	60.0 (13.6)	58.4 (16.2)	60.7 (12.7)	0.70
Sex				
Male	15 (45 %)	4 (40 %)	11 (48 %)	0.97
Female	18 (55 %)	6 (60 %)	12 (52 %)	
Weight (kg)	72.1 (12.9)	75.0 (14.1)	70.9 (12.4)	0.44
APACHE II score	19.9 (6.9)	19.0 (5.8)	20.4 (7.5)	0.58
Died in hospital				
Yes	16 (48 %)	7 (70 %)	9 (39 %)	0.21
No	17 (52 %)	3 (30 %)	14 (61 %)	
Admission to PLR (h)	26 [18, 52]	38 [21,102]	26 [16, 42]	0.25
Arrhythmia				
Present	3/32 (9 %)	1/9 (11 %)	2/23 (9 %)	1.00
Absent	29/32 (91 %)	8/9 (89 %)	21/23 (91 %)	
Tidal volume (ml)	510 [463, 563]	540 [473, 660]	491 [460, 549]	0.39
Plateau pressure (cmH_2_O)	26.6 (7.1)	26.4 (5.5)	26.7 (7.7)	0.90
PEEP (cmH_2_O)	8.5 (2.5)	8.9 (2.8)	8.4 (2.4)	0.60
Respiratory rate (/min)	16.8 (4.2)	15.5 (2.4)	17.3 (4.7)	0.15
Spontaneous breathing				
Yes	6/30 (20 %)	2/9 (22 %)	4/21 (19 %)	1.00
No	24/30 (80 %)	7/9 (78 %)	17/21 (81 %)	
Arterial line site:				
Femoral	4 (12 %)	1 (10 %)	3 (13 %)	1.00
Other (radial and brachial)	29 (88 %)	9 (90 %)	20 (87 %)	
Diagnostic groups:				
Septic shock	27 (82 %)	7 (70 %)	20 (87 %)	0.34
– Endocarditis	1 (3 %)	0 (0 %)	1 (4 %)	–
– Occult	7 (21 %)	2 (20 %)	5 (22 %)	–
– Peritonitis	8 (24 %)	2 (20 %)	6 (26 %)	–
– Pneumonia	11 (33 %)	3 (30 %)	8 (35 %)	–
SIRS	6 (18 %)	3 (30 %)	3 (13 %)	–
– Ischaemic bowel	2 (6 %)	1 (10 %)	1 (4 %)	–
– Occult	1 (3 %)	0	1 (4 %)	–
– Pancreatico-duodenectomy	1 (3 %)	0	1 (4 %)	–
– Pancreatitis	2 (6 %)	2 (20 %)	0	–

Data are shown as mean (standard deviation), median (inter-quartile range), or number (percentage) with *p* value comparing responders to non-responders for certain characteristics. Sample sizes are as in column headings unless stated (some variables had small numbers of missing values)

### Baseline haemodynamic variables and vasoactive infusions

There were no statistically significant differences in baseline haemodynamic variables (Table 2). Vasoactive drug infusions (Table 3) were similar between groups, and only two patients did not receive any vasoactive infusions at the time of the PLR test. Thirty (91 %) patients received a norepinephrine infusion with a mean dose of 0.30 (0.17) mcg/kg/min. Seven patients received Dobutamine, and this was split between responders and non-responders. Vasopressin infusions were used in two patients. It was combined with norepinephrine in one patient and used as sole vasopressor in the other.

**Table 2:** Haemodynamic variables at baseline

	All	Responders	Non-responders	*p*
	*n* = 33	*n* = 10	*n* = 23	
HR (/min)	100 (19.5)	106 (19.9)	98 (19.2)	0.29
CVP (mmHg)	10.6 (5.7)	9.9 (6.1)	10.9 (5.7)	0.66
MAP (mmHg)	69.5 (10.7)	65.1 (8.8)	71.5 (11.0)	0.09
PP (mmHg)	52.6 (13.1)	51.6 (17.8)	52.9 (11.2)	0.85
SV (ml/beat)	60.8 (14.5)	56.7 (16.3)	62.7 (13.7)	0.33
SVI (ml/m^2^/beat)	33.4 (7.8)	30.9 (8.8)	34.4 (7.4)	0.28
CO (L/min)	6.1 (1.5)	6.0 (1.6)	6.1 (1.5)	0.84
CI (L/min/m^2^)	3.3 (0.8)	3.3 (1.0)	3.4 (0.8)	0.81
SVV (%)	14.2 (11.0)	18.4 (10.5)	12.3 (11.0)	0.15
SVR (dynes s/cm^5^)	842 (347)	917 (504)	810 (259)	0.54

Data are shown as mean (standard deviation) or number (percentage) with *p* value comparing responders to non-responders.

*HR* heart rate, *CVP* central venous pressure, *MAP* mean arterial pressure, *PP* pulse pressure, *SV* stroke volume, *SVI* stroke volume index, *CO* cardiac output, *CI* cardiac index, *SVV* stroke volume variation, *SVR* systemic vascular resistance

**Table 3:** Vasoactive drug infusions

	All	Responders	Non-responders	*p*
Norepinephrine (mcg/kg/min)	0.28 (0.20, 0.36) (*n* = 30)	0.31 (0.16, 0.37) (*n* = 10)	0.28 (0.21, 0.34) (*n* = 20)	0.92
Vasopressin (mcg/min)	–	4.5 (*n* = 1)	3.0 (*n* = 1)^a^	–
Dobutamine (mcg/kg/min)	4.6 (1.2) (*n* = 7)	4.4 (1.3) (*n* = 4)	4.8 (1.2) (*n* = 3)	–

Data are shown as mean (standard deviation) or median (inter-quartile range), and number of patients (*n*) (*p* value compares dose between responders and non-responders in the case of norepinephrine). In the case of vasopressin, the only dose is given, as there was one patient only in each group

^a^The only patient requiring vasopressor support that did not include norepinephrine

### Effects of PLR and VE on CO and other haemodynamic variables

**Fig. 2:**
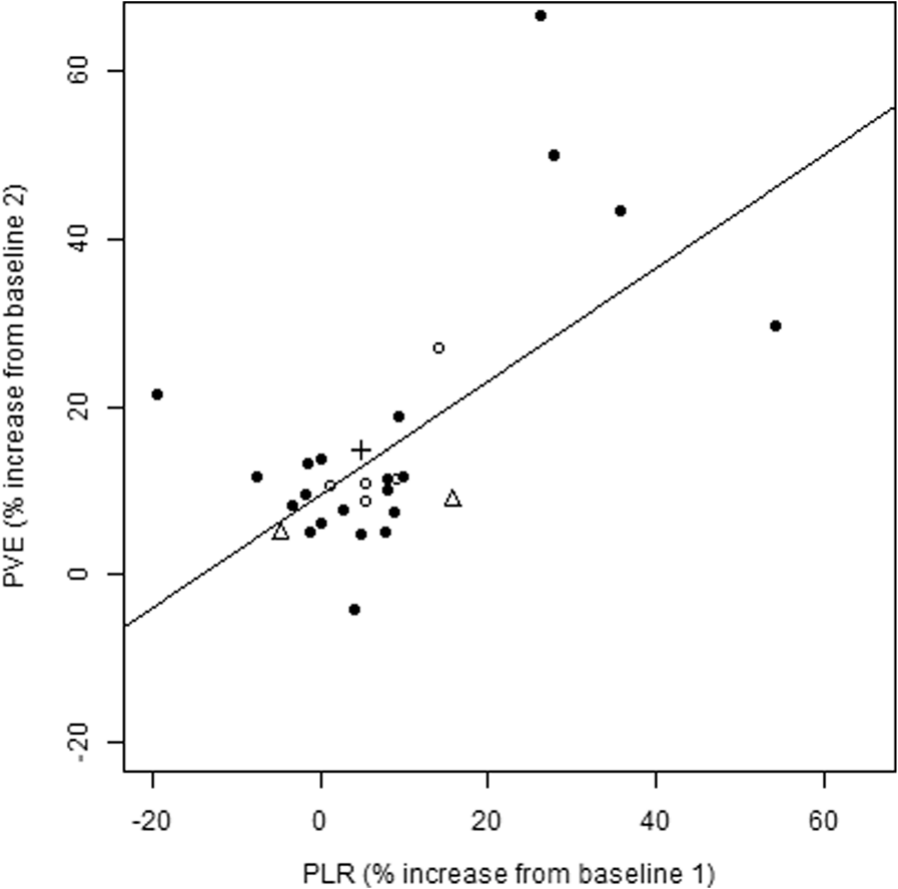
Relationship between percentage increase in cardiac output from baseline 1 after PLR and PVE after baseline 2 and line of best fit (correlation = 0.65). *Open circles* are patients with spontaneous breathing efforts; *triangles* are patients with arrhythmias; *crosses* are patients with both spontaneous breathing efforts and arrhythmias, and *black circles* are patients with neither spontaneous breathing efforts nor arrhythmias

The peak increase in CO occurred within 120 s in all cases. CO change during PLR was positively related to subsequent VE (correlation = 0.65) (Fig. 2). Spontaneous breathing and/or arrhythmia was only present in a few patients in each group thus not allowing any meaningful analysis of the influence of these two factors on the association (Table 1).

### Effects of PLR and VE on changes in CO

The changes in CO induced by PLR were significantly greater in responders than in non-responders (*p* = 0.02). In fluid responders, CO increased by an average of 1.43 l/min (95 % CI 0.53 to 2.33) from baseline (average 5.98) to during PLR (average 7.41), corresponding to a 24 % increase from baseline (95 % CI 8 to 39 %), and by an average of 1.94 l/min (95 % CI 0.99 to 2.89) from before VE (average 5.39) to after VE (average 7.33), corresponding to a 35 % increase from baseline (95 % CI 24 to 47 %).

In fluid non-responders, CO increased by an average of 0.24 l/min (95 % CI 0.09 to 0.39) from baseline (average 6.10) to during PLR (average 6.34), corresponding to a 3 % increase from baseline (95 % CI 1 to 6 %), and by an average of 0.45 l/min (95 % CI 0.31 to 0.59) from before VE (average 5.94) to after VE (average 6.39), corresponding to a 7 % increase from baseline (95 % CI 5 to 10 %).

Seven patients had an insignificant reduction in their CO during PLR (Fig. 2).

### Prediction of fluid responsiveness

**Fig. 3:**
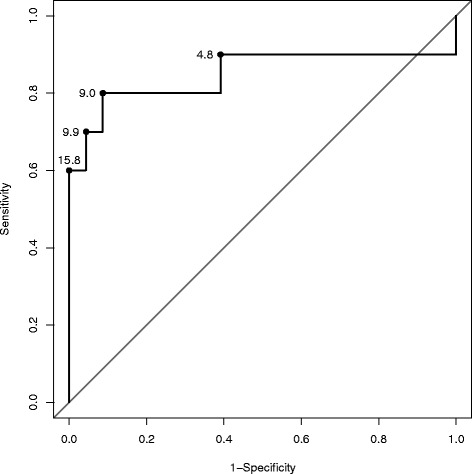
ROC curve demonstrating predictive performance after PLR on cardiac output response to PVE (AUC 0.85). Figures on the curve indicate the relevant cut point of cardiac output response (% change from baseline)

The ROC curve (Fig. 3) shows the varying predictive performance of the PLR test as the cut point changes. The optimal cut point on the ROC curve for this dataset was approximately 9 %, i.e. an increase in CO of 9 % or greater during PLR predicts fluid responsiveness with the greatest accuracy generating a sensitivity of 80 % (95 % CI 44 to 96 %) and a specificity of 91 % (95 % CI 70 to 98 %).

Although there was little distinction between a range of 10 to 15 % (for a 15 % cut point the sensitivity and specificity was 60 % (95 % CI 27 to 86 %) and 96 (95 % CI 76 to 99.8 %), the positive and negative predictive values were 86 % (695 % CI 42 to 99 %) and 85 % (95 % CI 64 to 95 %), respectively).

The area under the empirical ROC curve was 0.85 (95 % CI 0.63 to 1.00).

Using the increase in SVV during the PLR test as the predictor provides no better predictive performance than would be expected by chance with an area under the ROC curve of 0.56 (95 % CI 0.34 to 0.77).

An SVV cut point of 9.6 % at baseline, rather than change during PLR, as the predictor of fluid responsiveness, gives an area under the ROC curve of 0.74 (95 % CI 0.53 to 0.91), with 70 % sensitivity and 57 % specificity.

## Discussion

Our study shows that changes in CO measured using the Vigileo™ monitor during a PLR manoeuvre is a useful predictor of fluid responsiveness in mixed critically ill patients with vasopressor-dependent circulatory shock. An increase in CO of ≥ 9 % during the PLR predicted an increase in CO of ≥15 % following subsequent volume expansion with good sensitivity (80 %) and specificity (91 %) resulting in an AUC of 0.85 (95 % CI 0.63 to 1.00). The high specificity has the potential to avoid the deleterious effects of unnecessary volume expansion in this patient group [4, 6, 7, 11]*.*

Our results were consistent with previous validation studies of the PLR manoeuvre [21–38, 49] which are encapsulated in three recent systematic reviews [39–41]. These included nine, 21 and 23 studies assessing a total of 353, 991 and 1013, patients respectively. Our results were similar to the only other study using the Vigileo FloTrac device conducted by Biais et al. [14]. They reported a sensitivity of 85 % and a specificity of 90 % with a sample size of 34 patients and a peak change in flow within 120 s. Their study differed from our study in the following respects. They used second-generation FloTrac software whereas we used third-generation FloTrac software. Almost all the patients in the Biais study were surgical as opposed to an even split between medical and surgical in our study. The patients in our sample were much sicker, i.e. only 65 % were invasively ventilated in their study and all were breathing spontaneously with none receiving vasoactive drugs. That is in contrast with our study where all the patients were invasively ventilated and receiving vasoactive drugs (mean norepinephrine dosage was 0.3 ± 0.17mcg/kg/min). We reported a high APACHE2 score and hospital mortality.

Our study had the following limitations. Firstly, we identified less than one third of our sample as fluid responders (ten out of 33 subjects), whereas 50 % of patients were identified as responders in the systematic review by Michard et al. [3] and similarly in the PLR systematic reviews [39–41]. This may have weakened our calculated sensitivity and specificity. Responders were defined using a cut off of 15 %, as this definition was used consistently throughout the other published PLR studies [21–41, 49, 50], and we used the recommended PLR manoeuvre, i.e. patients started from a semi-recumbent position. This has been indentified as essential to ensure that splanchnic blood redistribution occurs thereby ensuring an adequate self-volume challenge [49].The small sample size and the volume chosen for our fluid challenges may have contributed to this lower rate of fluid responders. However, the sample size was still larger than half of all other PLR studies published [21–38, 49]. We chose 250 ml of Gelofusine for our fluid challenges as this was a pragmatic study which followed the local clinical practice at that time. Although most of the studies in the systematic reviews [39–41] used volumes of 500 ml, the study by Kang et al. [31] used 250 ml, and the study by Boulain et al. [21] used 300 ml of Gelatin. Both of these studies reported the usual rate of fluid responders and the Kang et al. study reported very high sensitivity and specificity. Boulain et al. discussed the rationale for choosing that volume, i.e. it is roughly equal to the volume of blood redistributed by the PLR manoeuvre. The volume and type of fluid we used were also consistent with the 2008 Surviving Sepsis Campaign Guidelines [51], a review by Vincent et al. [52] on circulatory shock and Trof et al. showed a more rapid change in SV with colloid boluses [53]. We delivered our volume challenge within fifteen minutes which is in keeping with the other studies.

Secondly, we measured CO using a single non-calibrated device and did not include a second calibrated device. However, the study by Biais et al. [28] had simultaneously measured haemodynamic changes during PLR with the Vigileo™ monitor and transthoracic echocardiography and found that the changes induced by volume expansion correlated well between these two devices (*r* ^2^ = 0.77, *p* < 0.0001). This was despite Biais et al. using the less accurate second-generation Vigileo software (version 1.14). In addition, several studies have validated the Vigileo third-generation software in the patient population we recruited. Meng et al. [46] assessed the trending ability of the Vigileo third-generation software using directly measured Oesophageal Doppler blood flow as the comparison device and found that the two devices showed 96 % concordance when measuring a change in preload induced by whole body tilting. De Backer et al. [45] reported acceptable accuracy using the third-generation software in patients with vasoplegia (septic shock and liver failure) as did Slagt et al. [47] in a systematic review comparing the different generations of Vigileo software. Despite the absence of a second calibrated device in our study, we derived a similar optimal cut point as the other published PLR studies [21–41, 49, 50] and specifically in the subgroups that also used pulse contour methods [28, 30–33].

Thirdly, in the majority of our patients (88 %), measurement was via a peripheral artery which may be less accurate than via a central artery in vasodilated patients [54]. The number receiving a central (femoral) arterial catheter was too few to analyse for this effect.

Finally, 39 % of the patients included in our study were surgical and therefore had the potential for intra-abdominal hypertension which has been shown by Mahjoub et al. [50] to reduce the accuracy of PLR for predicting fluid responsiveness. We did not have sufficient data on intra-abdominal pressures to analyse for this effect.

## Conclusions

We have demonstrated that changes in cardiac output measured using the Vigileo™ monitor with third-generation software during a PLR test were predictive of fluid responsiveness in both medical and surgical patients with vasopressor-dependent circulatory shock. As the EV1000™ monitoring system, which has replaced the Vigileo™ monitor, continues to use the third-generation software for the FloTrac device these findings remain valid.

## Additional file

Additional file 1: Study dataset. (CSV 8 kb)

## Abbreviations

AUC: Area under the receiving operating characteristic curve
CO: Cardiac output
PLR: Passive leg raise
PVE: Post volume expansion
SV: Stroke volume
TTE: Transthoracic echocardiography
VE: Volume expansion
